# Fibrosis-4 index as a predictor of all-cause and cardiovascular mortality in patients with chronic kidney disease

**DOI:** 10.1371/journal.pone.0329315

**Published:** 2025-08-01

**Authors:** Zheng-Yang Zhu, Ke-Jun Ren, Xiao-Wei Duan, Xu-Lei Hu, Yong Lv, Dong Wang, Hua Jin, Lei Zhang

**Affiliations:** 1 The First Clinical Medical College, Anhui University of Chinese Medicine, Hefei City, Anhui Province, China; 2 The First Affiliated Hospital, Anhui University of Chinese Medicine, Hefei City, Anhui Province, China; Cedars-Sinai Heart Institute, UNITED STATES OF AMERICA

## Abstract

This study utilized data from the National Health and Nutrition Examination Survey (NHANES) 2005–2020 (n = 28,231, including 4,907 chronic kidney disease (CKD) patients) with weighted analyses and, through variance inflation factor (VIF) assessment to verify covariate selection, supported model validity, to demonstrate for the first time that the Fibrosis-4 (FIB4) index serves as an independent predictor of all-cause and cardiovascular disease (CVD)-related mortality in CKD patients. Weighted logistic regression confirmed FIB4 index as a significant independent predictor of CKD risk (fully adjusted odds ratios (OR) 1.85, 95% confidence interval (CI) 1.64–2.08), with strong dose-response gradient (Q4 OR 3.51–6.02). Multivariable Cox proportional hazards regression models revealed that each 1-unit increase in the FIB4 index was associated with a 34% elevated risk of all-cause mortality (hazard ratio (HR) = 1.34, 95%CI: 1.27–1.41, p < 0.001) and a 34% increased risk of CVD mortality (HR = 1.34, 95% CI: 1.24–1.44, p < 0.001). Restricted cubic spline (RCS) analysis identified nonlinear threshold effects at inflection points of 1.84 (all-cause mortality) and 1.74 (CVD mortality), with mortality risks escalating sharply beyond these thresholds (all-cause HR = 2.57; CVD HR = 2.85). Receiver operating characteristic (ROC) curve analysis demonstrated robust predictive performance (area under the curve (AUC): 0.799 for all-cause mortality; 0.801 for CVD mortality). Subgroup analyses highlighted heightened risks among non-Hispanic Black individuals, older adults, and those with low physical activity. Mediation analysis indicated that neutrophil-to-lymphocyte ratio (NLR) mediated 5.48% of the FIB4–all-cause mortality association and 5.83% of the CVD mortality association, though direct effects predominated (94.52% and 94.17%, respectively). These findings establish the FIB4 index as a practical, evidence-based tool for risk stratification in CKD patients, offering critical insights for personalized clinical management.

## Introduction

The intersection of nephrology and cardiology, embodied in the emerging discipline of cardio-renal medicine, has gained critical importance amid the growing population of CKD patients and their heightened susceptibility to cardiovascular complications [1]. While advancements in renal replacement therapies and pharmacological interventions have improved survival rates, they have concurrently unmasked a spectrum of cardiovascular hazards inherent to CKD pathophysiology, including chronic inflammation, uremic cardiomyopathy, and accelerated atherosclerosis [2]. Although substantial progress has been made in stratifying cardiovascular risk in CKD populations, a persistent knowledge gap remains in identifying precise predictors or biomarkers for cardiovascular mortality within this vulnerable cohort. This unmet need underscores the urgency to develop innovative strategies for early risk prediction and targeted intervention in CKD-related cardiovascular pathologies.

The FIB4, a readily calculable composite marker incorporating age, platelet count, and hepatic transaminases (ALT/AST), has garnered attention for its dual role in evaluating hepatic fibrosis and systemic metabolic dysregulation [3]. Originally validated in hepatology, elevated FIB4 scores exhibit consistent associations with adverse outcomes across multiple organ systems [4], reflecting its capacity to capture subclinical inflammatory states and endothelial dysfunction. While its prognostic utility in predicting liver-related morbidity and all-cause mortality in metabolic syndrome populations is well-established, the potential of FIB4 to serve as a cardiovascular risk stratifier in CKD patients remains underexplored. This knowledge gap presents a compelling rationale for investigating FIB4’s capacity to bridge hepatic-renal-cardiovascular crosstalk, particularly given CKD’s characteristic interplay of chronic inflammation, oxidative stress, and multiorgan fibrotic remodeling.

Given the intricate interplay among inflammation, fibrosis, and multi-organ dysfunction, this study posits that the FIB4 index may serve as a pivotal biomarker for identifying heightened risks of all-cause and cardiovascular mortality in CKD patients. By leveraging longitudinal data from the NHANES, this retrospective cohort study aims to elucidate the association between FIB4 and both all-cause and cardiovascular-specific mortality in CKD populations, while exploring whether its underlying mechanisms are mediated through systemic inflammatory pathways reflected by the NLR. As a composite marker capturing hepatic-renal metabolic dysregulation and fibrotic progression, elevated FIB4 may exacerbate clinical outcomes in CKD through dual mechanisms: First, FIB4 directly quantifies hepatic and renal fibrosis burden, which pathologically synergizes with cardiovascular manifestations such as arterial stiffness and myocardial fibrosis [5]. Second, the significant correlation between FIB4 and NLR (both indicators of chronic inflammation) suggests that FIB4 may activate neutrophil-driven inflammatory cascades [6], promoting endothelial dysfunction and thrombogenesis, thereby accelerating the cardiovascular event cascade in CKD. This investigation not only addresses the evidence gap regarding FIB4’s prognostic value in CKD but also provides a novel multidimensional risk stratification perspective—integrating FIB4 and inflammation (NLR) markers could refine mortality prediction [7] and advance pathology-driven precision interventions for CKD patients [8].

## Methods

### Study population and design

**Fig 1:**
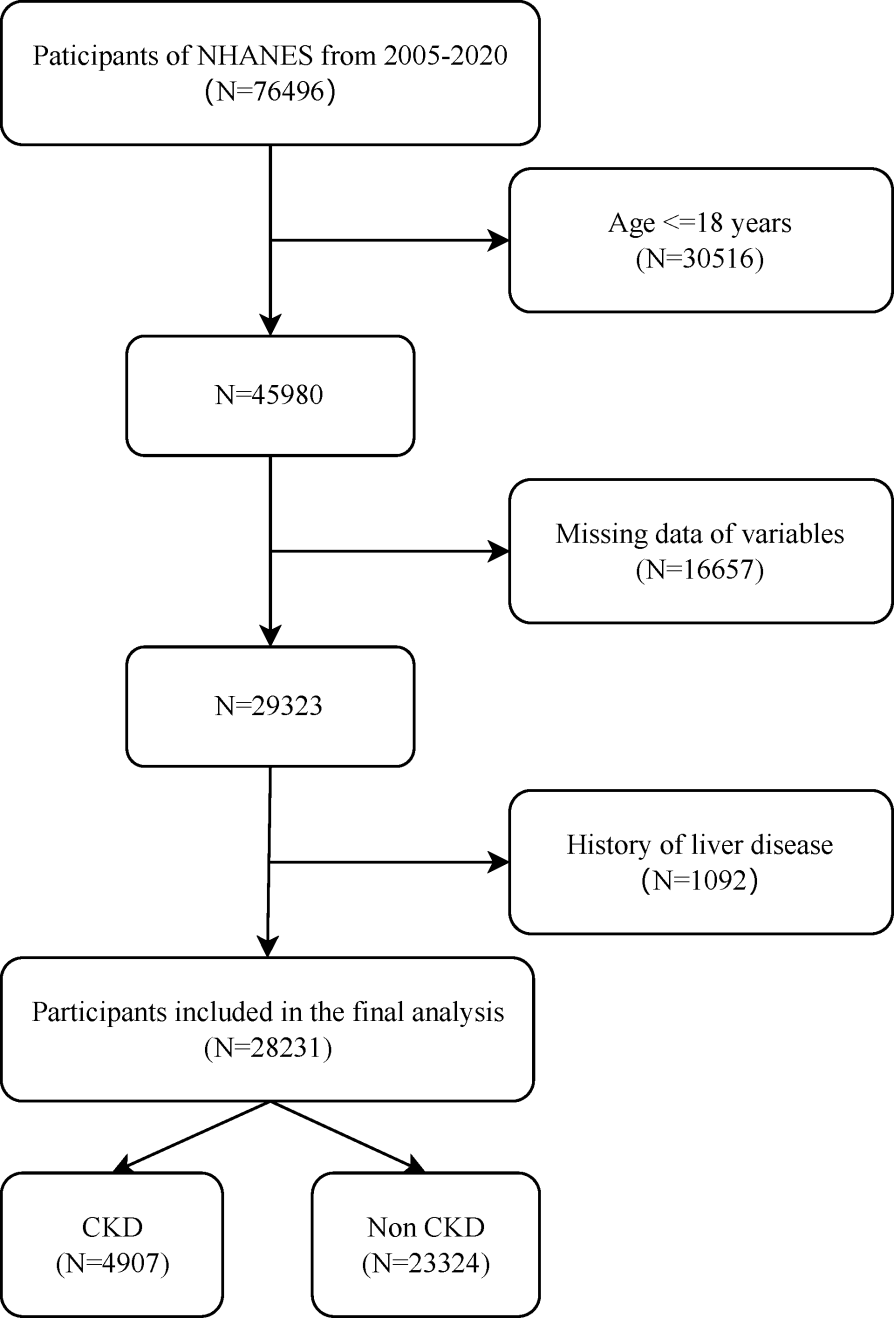
Flowchart of the study population (NHANES 2005–2020). After screening, a total number of 28231 participants were enrolled including 23324 controls (without CKD) and 4907 cases (with CKD). CKD, chronic kidney disease.

This study employed a hybrid cross-sectional and longitudinal cohort design using data from the NHANES (https://www.cdc.gov/nchs/nhanes), is an ongoing cross-sectional survey that evaluates the diet and health of the noninstitutionalized US civilian population [9], administered by the Centers for Disease Control and Prevention (CDC). NHANES utilizes a multistage probability sampling design to collect nationally representative health data through standardized protocols, including structured interviews, systematic physical examinations, 24-hour dietary recalls, and comprehensive laboratory assessments (serum biochemistry and urinalysis). From the publicly available NHANES 2005–2020 datasets, we applied sequential exclusion criteria to 76,496 initial participants to establish an adult cohort with comprehensive covariate profiles. This approach enabled us to rigorously examine whether, and to what extent, FIB4 index—as a potential systemic risk marker independent of advanced liver disease—is associated with CKD progression and risks of all-cause and CVD mortality: (1) age < 18 years (n = 30,516); (2) missing survival outcomes or critical covariates (n = 16,657); (3) comorbid viral hepatitis, cirrhosis, or active liver diseases (n = 1,092). The final analytical cohort comprised 28,231 eligible individuals (Fig 1). The research followed the tenets of the Declaration of Helsinki. Ethical considerations were rigorously followed, with all data sourced from the publicly available NHANES, which obtained informed consent from all participants and received ethical approval from the NCHS Research Ethics Review Board.

### Assessment of FIB4 and CKD

CKD was defined according to the Kidney Disease: Improving Global Outcomes (KDIGO) criteria as either [10]: (i) estimated glomerular filtration rate (eGFR) <60 mL/min/1.73 m² calculated using the CKD Epidemiology Collaboration (CKD-EPI [11]) creatinine equation (Equation 1); (ii) urinary albumin-to-creatinine ratio (UACR) ≥30 mg/g; or (iii) both. Laboratory analyses followed standardized protocols: the FIB4 index was calculated as FIB4 = [Age (years) × aspartate transaminase (AST) (μkat/L)]/ [Platelet count (Plt) (10⁹/L) × √ alanine transaminase (ALT) (μkat/L)]; eGFR was derived from the CKD-EPI; UACR was determined as urinary albumin (mg/L) divided by urinary creatinine (g/L) [12]; and the NLR was defined as absolute neutrophil count (10⁹/L) divided by absolute lymphocyte count (10⁹/L) [13].

### Determination of mortality

All-cause mortality and CVD-specific mortality were ascertained through linkage with the National Death Index (NDI) records (available at: https://www.cdc.gov/nchs/data-linkage/mortality-public.htm). Follow-up duration was calculated from the NHANES examination date to either the date of death or December 31, 2019, whichever occurred first. CVD mortality was defined as deaths attributed to underlying causes categorized under the International Classification of Diseases, Tenth Revision (ICD-10) codes I00–I09, I11, I13, I20–I51, or I60–I69, encompassing both cardiovascular and cerebrovascular diseases [14]. For conciseness, CVD mortality may be collectively referred to as cardiovascular mortality. According to the 2019 Public Use Linked Mortality Files code manual from the National Center for Health Statistics (NCHS), CVD mortality was operationally identified by the “ucode_leading” field values 001 and 005 in death records. The final cohort (N = 28,231) included 4,907 CKD patients and 23,324 non-CKD controls. The entire cohort was used for cross-sectional prevalence analyses, while multivariable Cox proportional hazards models were applied to the CKD subgroup (n = 4,907) to evaluate associations between FIB4 and risks of all-cause and cardiovascular-specific mortality.

eGFR = 141*min( $\frac{Scr}{\kappa }$ *,1)* ^*α*^ *max( $\frac{Scr}{\kappa }$ *, 1)* ^*−1.20*^ *0.993 ^*Age*^ * Sex Factor * Race Factor

**Equation 1** *Scr: Serum creatinine (mg/dL). κ: 0.7 (female), 0.9 (male). α: −0.329 (female), −0.411 (male). Sex factor: 1.018 (female), 1 (male). Race factor: 1.159 (black individuals), 1 (others).*

### Covariates

The analyses of all-cause mortality and cardiovascular mortality were adjusted for the same set of covariates. Participant characteristics encompassed age(<=60 years or >60 years), sex(male or female), ethnicity (categorized as Mexican American, Other Hispanic, Non-Hispanic White, Non-Hispanic Black, or Other Race), poverty-to-income ratio (PIR)(poor or not poor), educational level (classified as less than high school, high school or equivalent, or college and above) [15], smoking status (never or current smoker) [16], alcohol consumption status (never or current drinker), physical activity level (low or high), and marital status (married vs. other, including widowed, divorced, separated, never married, or cohabiting). CVD history was ascertained through self-reported physician diagnoses obtained via standardized medical condition questionnaires during in-person interviews. Participants were explicitly asked: “Has a physician or healthcare professional ever informed you that you have heart failure/chronic coronary artery disease/angina/myocardial infarction/stroke?” Affirmative responses to any of these conditions resulted in classification as CVD-positive. Hypertension was defined as systolic blood pressure >=18.7 kPa (140 mmHg), diastolic blood pressure >=12.0 kPa (90 mmHg), self-reported physician-diagnosed hypertension, or current use of antihypertensive medications [17]. Diabetes mellitus was diagnosed according to American Diabetes Association criteria, incorporating self-reported diagnoses, insulin/oral hypoglycemic agent use, fasting blood glucose >=7.0 mmol/L (126 mg/dL), random blood glucose >=11.1 mmol/L (200 mg/dL), or oral glucose tolerance test results >=11.1 mmol/L (200 mg/dL).Additional clinical parameters included fasting blood glucose (FBG), Plt, segmented neutrophil count (Segne), lymphocyte count (Lym), ALT, AST, serum albumin (ALB), uric acid (UA), triglycerides (TG), body mass index (BMI), high-density lipoprotein cholesterol (HDL), eGFR, UACR, and NLR.

### Statistical analysis

Given the complex sampling design of NHANES, all analyses incorporated sample weights, clustering, and stratification to comply with recommended analytic guidelines for NHANES data [18]. Participants were categorized into quartiles (Q1-Q4) based on FIB4 index values, with FIB4 quartiles defined as follows: Q1: FIB4 < 0.63; Q2: 0.63<=FIB4 < 0.92; Q3: 0.92<=FIB4 < 1.35; Q4: FIB4>=1.35. Continuous variables were summarized as weighted means with standard deviation (SD), while categorical variables were presented as frequencies with percentages. Intergroup comparisons of baseline characteristics were performed using one-way ANOVA with sample weighting for continuous variables and Pearson’s chi-square test with Rao-Scott adjustment for categorical variables. Since the calculation of FIB4 incorporates the age variable, age was not included in the models when performing weighted logistic regression, Cox regression, and other analyses.

Logistic regression models with sample weighting were employed to calculate adjusted OR for CKD risk between health status groups [19]. RCS with three knots [20], generalized additive models (GAM), and smooth curve fitting techniques were implemented to explore non-linear relationships between FIB4 and mortality outcomes, with adjustment for sex, ethnicity, marital status, PIR, education level, smoking status, alcohol consumption, physical activity, hypertension, and diabetes history. Optimal FIB4 thresholds for predicting all-cause and CVD mortality in CKD patients were determined through maximum likelihood estimation.

To minimize potential bias from complex survey design, weighted Cox proportional hazards models were constructed to assess the independent predictive value of FIB4 [21]. Three progressively adjusted models were developed: Model 1: unadjusted; Model 2: adjusted for ethnicity and sex; Model 3: We addressed multicollinearity among study variables and performed covariate selection using VIF. A VIF value exceeding 5 conventionally indicates problematic collinearity. Variables including sex, ethnicity, education level, marital status, PIR, drinking, smoking, physical activity, diabetes mellitus, and hypertension demonstrated calculated VIFs of less than 5. Therefore, all were retained for adjustment in Model 3 (S8 and S9 Tables). HR with 95% confidence intervals were calculated. The proportional hazards assumption was verified using Schoenfeld residual tests (all p > 0.05). To evaluate effect modification by prespecified subgroups (sex, age, ethnicity, education level, marital status, PIR, drinking, smoking, physical activity, diabetes mellitus, and hypertension), we incorporated interaction terms (FIB4 × subgroup) into weighted Cox proportional hazards models. Statistical significance of interactions was assessed using likelihood ratio tests comparing models with versus without interaction terms

Survival probabilities were estimated using Kaplan-Meier methods with survey weighting [22], and between-group differences were assessed via weighted log-rank tests. Predictive performance was evaluated through time-dependent ROC analysis using inverse probability weighting [23], with integrated AUC calculated at 5-, 10-, and 15- year intervals. Mediation analysis using counterfactual framework was performed to quantify the proportion of mortality risk mediated through NLR.

All analyses were conducted using R statistical software (version 4.3.2) with specialized packages: “survey” for complex sample analysis, “survival” for Cox models, “timeROC” for time-dependent AUC calculations, and “mediation” for pathway analysis. Statistical significance was defined as two-tailed p < 0.05. Data visualization was implemented using ggplot2 and survminer packages [24].

## Results

### Characteristics of the study population

As shown in S1 Table, this retrospective cohort study included 28,231 participants, represents 194,200,390 Americans (4,907 CKD patients, represents 26947641 Americans; 23,324 non-CKD controls). The cohort comprised 14,570 females (51.88%) and 13,661 males (48.12%), with 8,925 participants (24.61%) aged >60 years. Compared with the non-CKD group, the CKD group exhibited significant differences in multiple variables, including lower albumin (ALB: 4.19 vs. 4.31, p < 0.001), higher serum creatinine (Scr: 98.41 vs. 74.99, p < 0.001), and elevated uric acid (UA: 5.96 vs. 5.31, p < 0.001). Among the analyzed variables, only HDL (1.38 vs. 1.39, p = 0.513) showed no significant difference. CKD patients had significantly higher prevalence rates of hypertension (65.87% vs. 31.06%, p < 0.001), diabetes mellitus (33.64% vs. 9.86%, p < 0.001), and older age (>60 years: 59.00% vs. 19.07%, p < 0.001). The CKD group also included higher proportions of individuals with lower educational attainment (e.g., 23.26% with less than high school vs. 15.65%, p < 0.001), lower socioeconomic status (14.75% poor vs. 13.00%, p = 0.08), higher smoking rates (48.70% vs. 44.03%, p < 0.001), and low physical activity (52.89% vs. 35.06%, p < 0.001).

The FIB4 quartiles (FIB4Q) are described, and the general FIB4 quartile data are summarized in Table 1. The incidence of CKD among FIB4Q groups was Q1: 5.35%, Q2: 9.20%, Q3: 14.26%, and Q4: 23.83%; the CKD incidence significantly increased with higher FIB4 levels (Q1 to Q4: 5.35% → 23.83%, p < 0.001). With increasing FIB4 levels, multiple variables showed significant upward trends, including metabolic parameters (BMI: 29.05 vs. 28.32, p < 0.001; UA: 5.25 vs. 5.62, p < 0.001), inflammatory markers (NLR: 2.13 vs. 2.37, p < 0.001), renal injury indicators (UACR: 21.81 vs. 44.45, p < 0.001; Scr: 72.60 vs. 86.17, p < 0.001), and the prevalence of comorbid conditions (hypertension: Q1 16.77% vs. Q4 59.03%, p < 0.001; diabetes: Q1 5.35% vs. Q4 23.83%, p < 0.001). Populations characterized by older age (>60 years: 69.29% in Q4 vs. 0.39% in Q1, p < 0.001), female gender (48.35% in Q4 vs. 56.11% in Q1, p < 0.001), non-Hispanic Black ethnicity (9.44% in Q4 vs. 12.18% in Q1, p < 0.001), lower socioeconomic status (poor: 9.79% in Q4 vs. 20.36% in Q1, p = 0.08), and lower educational attainment (less than high school: 18.68% in Q4 vs. 16.55% in Q1, p < 0.001) were substantially more represented in the highest FIB4Q group (Q4).

**Table 1:** Weighted baseline characteristics of participants grouped by FIB4 quartile.

Variable	Total (n = 28231)	Q1 (n = 6650)	Q2 (n = 6565)	Q3 (n = 6878)	Q4 (n = 8138)	Statistic	*P*
ALT, Mean (SD)	25.28 (0.14)	24.85 (0.24)	25.26 (0.26)	25.06 (0.19)	25.95 (0.37)	F = 5.42	0.022
AST, Mean (SD)	25.42 (0.11)	22.20 (0.11)	23.96 (0.13)	25.30 (0.13)	30.23 (0.34)	F = 583.42	<.001
TG, Mean (SD)	152.91 (1.31)	141.45 (2.18)	157.07 (2.02)	159.62 (2.09)	153.51 (1.88)	F = 22.19	<.001
UA, Mean (SD)	5.40 (0.01)	5.25 (0.02)	5.31 (0.02)	5.41 (0.02)	5.62 (0.02)	F = 159.84	<.001
Scr, Mean (SD)	78.24 (0.23)	72.60 (0.31)	75.72 (0.33)	78.48 (0.38)	86.17 (0.54)	F = 461.69	<.001
FBG, Mean (SD)	5.59 (0.01)	5.35 (0.01)	5.51 (0.02)	5.66 (0.02)	5.83 (0.02)	F = 842.47	<.001
Lym, Mean (SD)	2.13 (0.01)	2.35 (0.01)	2.19 (0.01)	2.08 (0.02)	1.90 (0.01)	F = 776.52	<.001
Segne, Mean (SD)	4.32 (0.02)	4.72 (0.03)	4.41 (0.03)	4.18 (0.03)	3.97 (0.03)	F = 379.08	<.001
Plt, Mean (SD)	249.39 (0.79)	291.90 (1.27)	260.12 (0.94)	241.34 (1.05)	204.21 (0.82)	F = 3433.97	<.001
HDL, Mean (SD)	1.39 (0.01)	1.31 (0.01)	1.36 (0.01)	1.41 (0.01)	1.47 (0.01)	F = 210.51	<.001
Uscr, Mean (SD)	120.55 (0.89)	136.99 (1.48)	123.27 (1.44)	113.30 (1.22)	108.64 (1.23)	F = 250.29	<.001
BMI, Mean (SD)	28.78 (0.08)	29.05 (0.15)	28.87 (0.14)	28.89 (0.11)	28.32 (0.11)	F = 15.93	<.001
UACR, Mean (SD)	31.30 (1.44)	21.81 (2.85)	29.56 (3.52)	29.39 (2.78)	44.45 (2.86)	F = 29.15	<.001
eGFR, Mean (SD)	99.86 (0.30)	116.17 (0.35)	105.81 (0.33)	95.25 (0.43)	82.20 (0.38)	F = 4241.75	<.001
NLR, Mean (SD)	2.20 (0.01)	2.13 (0.01)	2.14 (0.02)	2.18 (0.02)	2.37 (0.02)	F = 81.33	<.001
Sex, n (%)						χ² = 86.56	<.001
Male	13661 (48.12)	2761 (43.89)	3070 (48.27)	3335 (48.66)	4495 (51.65)		
Female	14570 (51.88)	3889 (56.11)	3495 (51.73)	3543 (51.34)	3643 (48.35)		
Ethnicity, n (%)						χ² = 698.95	<.001
Mexican American	4617 (8.56)	1377 (12.66)	1170 (10.09)	1124 (7.20)	946 (4.28)		
Other Hispanic	2695 (5.29)	658 (7.25)	672 (6.18)	692 (4.54)	673 (3.21)		
Non-Hispanic White	12271 (68.17)	2567 (60.13)	2631 (64.17)	2902 (71.07)	4171 (77.32)		
Non-Hispanic Black	5846 (10.87)	1341 (12.18)	1322 (11.15)	1497 (10.69)	1686 (9.44)		
Other Race	2802 (7.10)	707 (7.77)	770 (8.41)	663 (6.49)	662 (5.75)		
Marital status, n (%)						χ² = 464.36	<.001
Married	17103 (64.38)	3485 (54.00)	4245 (67.65)	4484 (69.82)	4889 (66.05)		
Other (widowed, divorced, Separated, never married, living with a partner)	11128 (35.62)	3165 (46.00)	2320 (32.35)	2394 (30.18)	3249 (33.95)		
PIR, n (%)						χ² = 459.69	<.001
Poor	5513 (13.24)	1764 (20.36)	1279 (13.09)	1160 (9.74)	1310 (9.79)		
Not Poor	22718 (86.76)	4886 (79.64)	5286 (86.91)	5718 (90.26)	6828 (90.21)		
Smoking, n (%)						χ² = 110.52	<.001
No	15767 (55.32)	4124 (59.48)	3813 (56.33)	3718 (54.61)	4112 (50.87)		
Yes	12464 (44.68)	2526 (40.52)	2752 (43.67)	3160 (45.39)	4026 (49.13)		
Education level, n (%)						χ² = 61.52	<.001
Less than high school	7192 (16.70)	1467 (16.55)	1476 (15.39)	1839 (16.20)	2410 (18.68)		
high school or equivalent	6448 (22.48)	1572 (22.88)	1401 (21.12)	1516 (22.00)	1959 (23.93)		
college or above	14591 (60.81)	3611 (60.57)	3688 (63.48)	3523 (61.80)	3769 (57.39)		
Drinking, n (%)						χ² = 49.20	<.001
No	9738 (28.65)	2164 (27.20)	2169 (27.73)	2390 (27.75)	3015 (31.89)		
Yes	18493 (71.35)	4486 (72.80)	4396 (72.27)	4488 (72.25)	5123 (68.11)		
Physical activity, n (%)						χ² = 199.18	<.001
Low physical activity	11849 (37.53)	2431 (33.71)	2426 (34.69)	2942 (37.61)	4050 (44.13)		
High physical activity	16382 (62.47)	4219 (66.29)	4139 (65.31)	3936 (62.39)	4088 (55.87)		
Hypertension, n (%)						χ² = 3096.28	<.001
No	16866 (64.11)	5557 (83.23)	4684 (73.21)	3721 (59.06)	2904 (40.97)		
Yes	11365 (35.89)	1093 (16.77)	1881 (26.79)	3157 (40.94)	5234 (59.03)		
Diabetes mellitus, n (%)						χ² = 1183.87	<.001
No	23261 (86.84)	6237 (94.65)	5769 (90.80)	5512 (85.74)	5743 (76.17)		
Yes	4970 (13.16)	413 (5.35)	796 (9.20)	1366 (14.26)	2395 (23.83)		
Age, n (%)						χ² = 11425.12	<.001
<=60	19306 (75.39)	6622 (99.61)	6174 (95.90)	4648 (75.33)	1862 (30.71)		
> 60	8925 (24.61)	28 (0.39)	391 (4.10)	2230 (24.67)	6276 (69.29)		

All estimates accounted for complex survey designs. Values are presented as mean ± SD for continuous variables, and P-value was calculated by the weighted linear regression. Values are presented as percent (%) for categorical variables, and P-value was calculated by weighted chi-square test.

ALB: albumin; ALT: alanine aminotransferase; AST: aspartate aminotransferase; TG: triglycerides; UA: uric acid; Scr: serum creatinine; FBG: fasting blood glucose; Lym: lymphocyte count; Segne: segmented neutrophils; Plt: platelet count; HDL: high-density lipoprotein; Uscr: urinary creatinine; UACR: urinary albumin-to-creatinine ratio; EGFR: estimated glomerular filtration rate; NLR: neutrophil-to-lymphocyte ratio; BMI: body mass index; PIR: poverty-income ratio; SD: standard deviation; HR: hazard ratio; CI: confidence interval; OR: odds ratio; t: Student’s t-test; χ²: chi-square test.

### The correlation between FIB4 and CKD incidence

This multivariable analysis demonstrated that FIB4 was significantly associated with CKD risk (Table 2), with an unadjusted OR of 2.46 (95% CI: 2.20–2.75, P < 0.001) in Model 1. The association remained significant after adjustments in Model 2 (OR=2.52, 95% CI: 2.25–2.84, P = 0.002) and Model 3 (OR=1.85, 95% CI: 1.64–2.08, P < 0.001). FIB4Q analysis revealed a dose-dependent risk gradient in Model 1: Q2 had OR=1.25 (95% CI: 1.07–1.46, P = 0.006), Q3 had OR=1.90 (95% CI: 1.66–2.18, P < 0.001), and Q4 had OR=6.02 (95% CI: 5.35–6.77, P < 0.001). In Model 2, the corresponding ORs were Q2: 1.28 (95% CI: 1.10–1.50, P = 0.002); Q3: 1.97 (95% CI: 1.72–2.26, P < 0.001); Q4: 6.42 (95% CI: 5.70–7.24, P < 0.001). After adjustment in Model 3, Q2 became nonsignificant (OR=1.10, 95% CI: 0.94–1.29, P = 0.237), while Q3 (OR=1.35, 95% CI: 1.17–1.57, P < 0.001) and Q4 (OR=3.51, 95% CI: 3.08–4.00, P < 0.001) retained significance. These findings confirm FIB4 as an independent CKD risk predictor, with persistent significance across adjusted models. The pronounced risk elevation in FIB4Q Q4 (6.02-fold in Model 1) underscores its clinical utility for CKD risk stratification.

**Table 2:** Logistic regression for FIB4, FIB4Q.

Variables	CKD					
	OR (95%CI)^a^	*P*	OR (95%CI)^b^	*P*	OR (95%CI)^c^	*P*
FIB4	2.46 (2.20 ~ 2.75)	<.001	2.52 (2.25 ~ 2.84)	0.002	1.85 (1.64 ~ 2.08)	<.001
FIB4Q						
Q1	1.00 (Reference)		1.00 (Reference)		1.00 (Reference)	
Q2	1.25 (1.07 ~ 1.46)	0.006	1.28 (1.10 ~ 1.50)	0.002	1.10 (0.94 ~ 1.29)	0.237
Q3	1.90 (1.66 ~ 2.18)	<.001	1.97 (1.72 ~ 2.26)	<.001	1.35 (1.17 ~ 1.57)	<.001
Q4	6.02 (5.35 ~ 6.77)	<.001	6.42 (5.70 ~ 7.24)	<.001	3.51 (3.08 ~ 4.00)	<.001

a: Model 1: unadjusted; b: Model 2: adjusted for sex and ethnicity; c: Model 3: Adjust: sex, ethnicity, PIR, smoking, drinking, physical activity, education level, hypertension, and diabetes mellitus.

FIB4: Fibrosis-4 Index; FIB4Q: Fibrosis-4 Index quartile; OR: odds ratio; CI: confidence interval; CKD: chronic kidney disease.

### FIB4 association with all-cause mortality and CVD mortality

**Fig 2:**
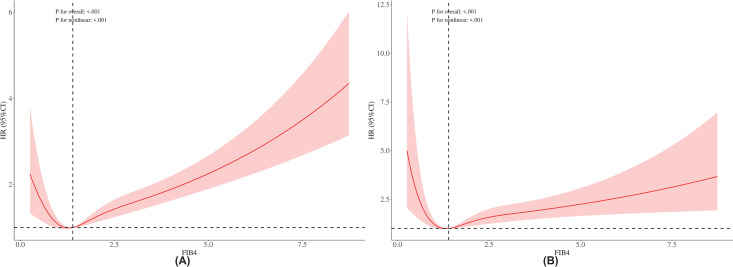
The associations between FIB4 and all-cause mortality (A) and CVD mortality (B) were visualized using restricted cubic splines. Panel A demonstrates the nonlinear association between FIB4 and all-cause mortality, while Panel B depicts the nonlinear association between FIB4 and CVD mortality. Hazard ratios were adjusted for sex, ethnicity, PIR, smoking, drinking, physical activity, education level, hypertension, and diabetes mellitus. HR: hazard ratio; CI: confidence interval; FIB4: Fibrosis-4 Index.

During a median follow-up of 84 months, among 4,907 CKD patients, cardiovascular-specific mortality (11.55%, n = 567) demonstrated significant associations with elevated Scr (116.22 vs. 96.55, P < 0.001) and reduced eGFR (: 8.72 vs. 77.47, P < 0.001). All-cause mortality (31.83%, n = 1,562) correlated with hyperglycemia (FBG: 6.21 vs. 6.08 mmol/L, P = 0.005), lower body mass index (BMI: 29.42 vs. 30.05, P = 0.037), smoking prevalence (56.43% vs. 45.86%, P < 0.001), and shared renal dysfunction (Scr: 116.12 vs. 91.90 μmol/L, P < 0.001). Elevated FIB4 (2.08 vs. 1.50, P < 0.001), male sex (47.12% vs. 41.35%, P < 0.05), Non-Hispanic White ethnicity (77.87% vs. 69.14%, P < 0.05), and unmarried status (51.37% vs. 40.29%, P < 0.05) independently predicted both mortality outcomes (S2 and S3 Tables). The RCS were applied to explore the nonlinear relationship and optimal thresholds between the FIB4 and all-cause mortality as well as CVD-specific mortality. The results revealed significant J-shaped associations for both outcomes (P for nonlinearity <0.001), with overall associations being statistically significant (P for overall <0.001). The inflection point (optimal threshold) of FIB4 for all-cause mortality was identified at 1.84 (Table 3), whereas a lower threshold of 1.74 was observed for CVD-specific mortality (Fig 2). Notably, mortality risks increased sharply when FIB4 levels exceeded these thresholds, highlighting the critical role of FIB4 in risk stratification for adverse clinical outcomes.

**Table 3:** Threshold analysis for FIB4 and mortality in CKD.

FIB4	Adjusted Model OR (95%CI)	*P*
All-cause mortality		
Model 1 Fitting model by standard linear regression	1.34 (1.30 - 1.38)	<.001
Model 2 Fitting model by two-piecewise linear regression		
Inflection point	1.839	
<1.839	3.65 (2.96 - 4.51)	<.001
≥1.839	1.16 (1.11 - 1.22)	<.001
P for likelihood test		<.001
CVD mortality		
Model 1 Fitting model by standard linear regression	1.17 (1.08 - 1.25)	<.001
Model 2 Fitting model by two-piecewise linear regression		
Inflection point	1.738	
< 1.738	4.61 (3.06 - 6.94)	<.001
≥ 1.738	1.16 (1.07 - 1.25)	<.001
P for likelihood test		<.001

OR, odds ratio; CI, confidence interval. Adjusted for sex, ethnicity, marital status, education level, smoking, drinking, physical activity, PIR, hypertension, and diabetes mellitus.

In the unadjusted model (Model 1), increased FIB4 values were significantly associated with higher risks of all-cause mortality and CVD mortality (all-cause mortality: HR 1.31, 95% CI 1.21–1.42, p < 0.001; CVD mortality: HR 1.30, 95% CI 1.19–1.43, p < 0.001). After adjusting for sex and ethnicity (Model 2), each unit increase in FIB4 was associated with a 30% elevated risk of all-cause mortality (HR 1.30, 95% CI 1.20–1.40, p < 0.001) and a 30% increased risk of CVD mortality (HR 1.30, 95% CI 1.18–1.42, p < 0.001). Further adjustment for additional covariates, including marital status, PIR, smoking, education level, drinking, physical activity, hypertension, and diabetes mellitus (Model 3), revealed that each unit increment in FIB4 corresponded to a 34% higher risk of all-cause mortality (HR 1.34, 95% CI 1.27–1.41, p < 0.001) and a 34% increased risk of CVD mortality (HR 1.34, 95% CI 1.24–1.44, p < 0.001). When FIB4 was categorized using thresholds, Model 3 analysis showed participants with FIB4 > 1.84 had 2.57-fold higher all-cause mortality risk versus ≤1.84 (HR = 2.57, 95% CI: 2.21–2.98, p < 0.001), while those with FIB4 > 1.74 had 2.85-fold higher CVD mortality risk versus ≤1.74 (HR = 2.85, 95% CI: 2.22–3.66, p < 0.001) (Table 4).

**Table 4:** The relationships between FIB4 and mortality in CKD.

Variables	Model1		Model2		Model3	
	HR (95%CI)	*P*	HR (95%CI)	*P*	HR (95%CI)	*P*
**All-cause mortality**						
FIB4	1.31 (1.21 - 1.42)	<.001	1.30 (1.20 - 1.40)	<.001	1.34 (1.27 - 1.41)	<.001
FIB4T1						
<= 1.84	1.00 (Reference)		1.00 (Reference)		1.00 (Reference)	
> 1.84	3.47 (3.07 - 3.91)	<.001	3.23 (2.85 - 3.66)	<.001	2.57 (2.21 - 2.98)	<.001
**CVD mortality**						
FIB4	1.30 (1.19 - 1.43)	<.001	1.30 (1.18 - 1.42)	<.001	1.34 (1.24 - 1.44)	<.001
FIB4T2						
<=1.74	1.00 (Reference)		1.00 (Reference)		1.00 (Reference)	
> 1.74	3.86 (3.10 - 4.81)	<.001	3.62 (2.90 - 4.52)	<.001	2.85 (2.22 - 3.66)	<.001

Data are presented as HR (95% CI). Model 1, unadjusted; Model 2, adjusted for sex and ethnicity; Model 3, adjusted for sex, ethnicity, marital status, PIR, smoking, education level, drinking, physical activity, hypertension, and diabetes mellitus.

FIB4: Fibrosis-4 Index; FIB4T1: The threshold effect of Fibrosis-4 Index for all-cause mortality; FIB4T2: The threshold effect of Fibrosis-4 Index for CVD mortality; HR: hazard ratio; CI: confidence interval; CVD: cardiovascular disease.

**Fig 3:**
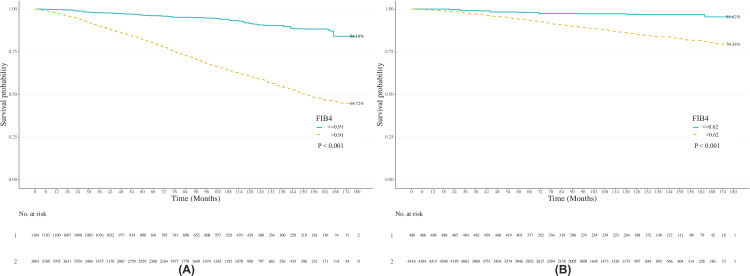
Kaplan-Meier analysis demonstrated the association between FIB4 and both all-cause mortality (A) and CVD mortality (B). Panel A displays the Kaplan-Meier analysis results for the association between FIB4 and all-cause mortality, while Panel B displays the Kaplan-Meier analysis results for the association between FIB4 and CVD mortality. FIB4: Fibrosis-4 Index.

Kaplan-Meier analysis further demonstrated that the high FIB4 group (all-cause mortality: 1.84; CVD mortality: > 1.74) exhibited significantly lower survival probabilities compared to the low FIB4 group (all-cause mortality: ≤ 1.84; CVD mortality: ≤ 1.74) (log-rank p < 0.001 for both outcomes) (Fig 3).

### Subgroup analyses of the association between FIB4 and mortality

**Fig 4:**
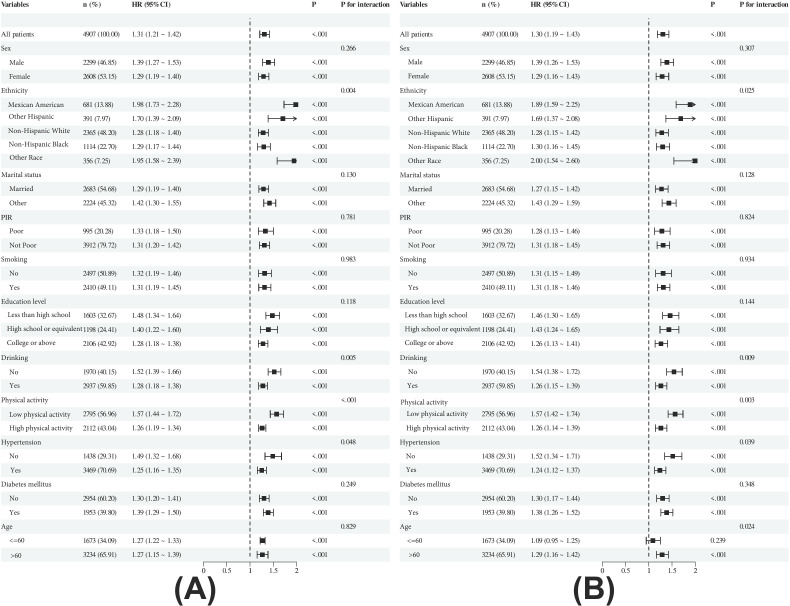
Subgroup and Interaction Analyses. (A) Subgroup analysis of the association between FIB4 and all-cause mortality. (B) Subgroup analysis of the association between FIB4 and CVD mortality. Subgroup analyses were performed using weighted Cox regression models. Forest plots were generated based on weighted Cox regression estimates. Interaction analyses were conducted using likelihood ratio tests. HR: hazard ratio; CI: confidence interval; PIR: poverty-income ratio.

Subgroup analysis forest plots (Fig 4) demonstrated that elevated FIB4 was an independent risk factor for both all-cause mortality and CVD mortality. For all-cause mortality, significant associations of FIB4 were observed in subgroups including Non-Hispanic Black individuals (HR 1.29, 95% CI 1.17–1.44), those with low physical activity (HR 1.57, 95% CI 1.44–1.72), hypertensive patients (HR 1.49, 95% CI 1.32–1.68), and individuals aged >60 years (HR 1.27, 95% CI 1.15–1.39) (all p < 0.001). For CVD mortality, elevated FIB4 was significantly associated with higher risks in “Other Race” subgroups (HR 2.00, 95% CI 1.54–2.60), low physical activity (HR 1.57, 95% CI 1.42–1.74), and age > 60 years (HR 1.29, 95% CI 1.16–1.42) (all p < 0.001). Interaction analyses revealed significant effect modifications between FIB4 and ethnicity (all-cause p = 0.004; CVD p = 0.025), physical activity (all-cause p < 0.001; CVD p = 0.005), and drinking status (all-cause p = 0.005; CVD p = 0.009), suggesting potential modulation of FIB4-mortality associations by these factors. However, the effect of FIB4 on mortality remained consistent across most subgroups (interaction p > 0.05), supporting its role as an independent risk factor. (Table 5).

**Table 5:** Subgroup analyses of the association between FIB4 and mortality in CKD patients.

	All-cause mortality			CVD mortality		
Variables	HR (95%CI)	*P*	P for interaction	HR (95%CI)	*P*	P for interaction
All patients	1.31 (1.21 ~ 1.42)	<.001		1.30 (1.19 ~ 1.43)	<.001	
Sex, n (%)			0.266			0.307
Male	1.39 (1.27 ~ 1.53)	<.001		1.39 (1.26 ~ 1.53)	<.001	
Female	1.29 (1.19 ~ 1.40)	<.001		1.29 (1.16 ~ 1.43)	<.001	
Ethnicity, n (%)			0.004			0.025
Mexican American	1.98 (1.73 ~ 2.28)	<.001		1.89 (1.59 ~ 2.25)	<.001	
Other Hispanic	1.70 (1.39 ~ 2.09)	<.001		1.69 (1.37 ~ 2.08)	<.001	
Non-Hispanic White	1.28 (1.18 ~ 1.40)	<.001		1.28 (1.15 ~ 1.42)	<.001	
Non-Hispanic Black	1.29 (1.17 ~ 1.44)	<.001		1.30 (1.16 ~ 1.45)	<.001	
Other Race	1.95 (1.58 ~ 2.39)	<.001		2.00 (1.54 ~ 2.60)	<.001	
Marital status, n (%)			0.130			0.128
Married	1.29 (1.19 ~ 1.40)	<.001		1.27 (1.15 ~ 1.42)	<.001	
Other (widowed, divorced, Separated, never married, living with a partner)	1.42 (1.30 ~ 1.55)	<.001		1.43 (1.29 ~ 1.59)	<.001	
PIR, n (%)			0.781			0.824
Poor	1.33 (1.18 ~ 1.50)	<.001		1.28 (1.13 ~ 1.46)	<.001	
Not Poor	1.31 (1.20 ~ 1.42)	<.001		1.31 (1.18 ~ 1.45)	<.001	
Smoking, n (%)			0.983			0.934
No	1.32 (1.19 ~ 1.46)	<.001		1.31 (1.15 ~ 1.49)	<.001	
Yes	1.31 (1.19 ~ 1.45)	<.001		1.31 (1.18 ~ 1.46)	<.001	
Education level, n (%)			0.118			0.144
Less than high school	1.48 (1.34 ~ 1.64)	<.001		1.46 (1.30 ~ 1.65)	<.001	
high school or equivalent	1.40 (1.22 ~ 1.60)	<.001		1.43 (1.24 ~ 1.65)	<.001	
college or above	1.28 (1.18 ~ 1.38)	<.001		1.26 (1.13 ~ 1.41)	<.001	
Drinking, n (%)			0.005			0.009
No	1.52 (1.39 ~ 1.66)	<.001		1.54 (1.38 ~ 1.72)	<.001	
Yes	1.28 (1.18 ~ 1.38)	<.001		1.26 (1.15 ~ 1.39)	<.001	
Physical activity, n (%)			<.001			0.003
Low physical activity	1.57 (1.44 ~ 1.72)	<.001		1.57 (1.42 ~ 1.74)	<.001	
High physical activity	1.26 (1.19 ~ 1.34)	<.001		1.26 (1.14 ~ 1.39)	<.001	
Hypertension, n (%)			0.048			0.039
No	1.49 (1.32 ~ 1.68)	<.001		1.52 (1.34 ~ 1.71)	<.001	
Yes	1.25 (1.16 ~ 1.35)	<.001		1.24 (1.12 ~ 1.37)	<.001	
Diabetes mellitus, n (%)			0.249			0.348
No	1.30 (1.20 ~ 1.41)	<.001		1.30 (1.17 ~ 1.44)	<.001	
Yes	1.39 (1.29 ~ 1.50)	<.001		1.38 (1.26 ~ 1.52)	<.001	
Age, n (%)			0.829			0.024
<=60	1.27 (1.22 ~ 1.33)	<.001		1.09 (0.95 ~ 1.25)	0.239	
> 60	1.27 (1.15 ~ 1.39)	<.001		1.29 (1.16 ~ 1.42)	<.001	

Data presented as adjusted HR and 95% CI. All HR were adjusted for sex, ethnicity, marital status, PIR, smoking status, education level, drinking, physical activity, hypertension, and diabetes mellitus.

PIR: poverty-to-income ratio; HR: hazard ratio; CI: confidence interval; CVD: cardiovascular disease.

### Sensitivity and specificity analysis

**Fig 5:**
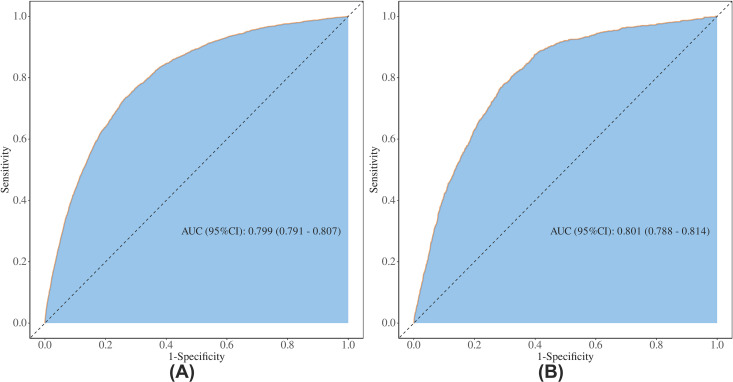
ROC curve analysis of the FIB4 for predicting outcomes in patients with CKD. (A) ROC curve analysis of the FIB4 for predicting all-cause mortality. (B) ROC curve analysis of the FIB4 for predicting CVD mortality. AUC, area under the curve; CI, confidence interval.

ROC curves were constructed to evaluate the sensitivity and specificity of FIB4 as a prognostic tool. The predictive capacity of FIB4 for all-cause mortality was substantial, with an AUC of 0.799 (95% CI: 0.791–0.807). Similarly, FIB4 demonstrated strong predictive performance for cardiovascular mortality, achieving an AUC of 0.801 (95% CI: 0.788–0.814) (Fig 5).

### Mediation analysis of the FIB4 and NLR

Mediation analyses indicated that the association between FIB4 and both all-cause mortality and CVD mortality was partially mediated by the NLR. After adjusting for covariates, the indirect effect accounted for 5.48% of the total effect for all-cause mortality (β = −1.48, 95% CI: −2.12 to −0.89, p < 0.001), while the direct effect explained 94.52% (β = −25.99, 95% CI: −33.61 to −18.69, p < 0.001). For CVD mortality, the indirect effect via NLR represented 5.83% (β = −1.54, 95% CI: −2.23 to −0.91, p < 0.001), with the direct effect contributing 94.17% (β = −24.89, 95% CI: −33.11 to −16.01, p < 0.001). Pathway analysis further demonstrated a statistically significant indirect effect of FIB4 on mortality through NLR (all-cause mortality: β = 0.12, 95% CI: 0.06–0.18, p < 0.001; CVD mortality: β = 0.12, 95% CI: 0.06–0.18, p < 0.001). Additionally, the direct effect of FIB4 on mortality remained robust (all-cause mortality: β = 0.26, 95% CI: 0.16–0.36, p < 0.001; CVD mortality: β = 0.26, 95% CI: 0.16–0.36, p < 0.001). These findings suggest that while NLR partially mediates the association between FIB4 and mortality, the direct effect of FIB4 on mortality remains predominant. (S4–S7 Tables).

## Discussion

The FIB4, a pivotal biomarker reflecting the equilibrium between hepatic fibrosis severity and renal function, exhibits elevated values indicative of progressive fibrogenesis and systemic metabolic dysregulation [25]. Our study provides robust evidence that FIB4 serves as a critical prognostic marker for cardiovascular mortality in patients with CKD [26], underscoring novel interactions within the hepatic-renal-cardiovascular axis [27]. Through comprehensive analysis of NHANES data, we elucidated the independent predictive capacity of elevated FIB4 levels in this population, with particular emphasis on cardiovascular mortality [28]. This finding represents a paradigm shift by extending the established utility of FIB4 in predicting liver fibrosis-related outcomes to its novel role in anticipating cardiovascular complications in CKD patients. Despite extensive documentation of multi-organ fibrotic effects in CKD pathophysiology, reliable biomarkers for predicting cardiovascular outcomes in this vulnerable population remain scarce. Our research addresses this gap by demonstrating that FIB4—a simple, readily calculable fibrosis marker—effectively identifies CKD individuals at heightened risk of cardiovascular mortality. This advancement not only enriches our understanding of the interplay between hepatic fibrosis, metabolic derangements, and cardiovascular disease but also paves the way for innovative preventive strategies and therapeutic interventions to reduce cardiovascular morbidity and mortality in CKD populations.

Notably, non-Hispanic Black individuals and elderly patients were disproportionately represented in high FIB4 quartiles, while those with lower socioeconomic status or comorbid hypertension/diabetes also demonstrated elevated FIB4 levels. These observations suggest complex interactions among sociodemographic factors, metabolic disturbances, and cardiovascular risk profiles in CKD patients [29]. Prior investigations have explored associations between FIB4 and multi-organ fibrosis, with emerging evidence linking elevated FIB4 levels to dysregulation of the hepatic-renal-cardiovascular axis. Our findings align with existing literature while uniquely emphasizing FIB4’s prognostic relevance for cardiovascular mortality in CKD. This consistency reinforces FIB4’s robustness as a systemic fibrosis marker associated with adverse outcomes across diverse chronic disease populations. Leveraging the NHANES dataset—renowned for its demographic diversity and national representativeness—enhanced the generalizability of our results, providing critical insights into FIB4’s utility for risk stratification in broad clinical contexts.

RCS analysis revealed nonlinear relationships between FIB4 and mortality endpoints (all-cause and cardiovascular), with risks escalating markedly beyond specific thresholds [30]. The identified inflection points (1.84 for all-cause mortality; 1.74 for cardiovascular mortality) highlight FIB4’s clinical utility, demonstrating exponential risk elevation beyond these cutoffs. These thresholds provide critical intervention nodes, emphasizing the necessity for intensified monitoring in CKD patients exceeding these values. The differential thresholds across mortality outcomes suggest distinct mechanistic pathways through which hepatic fibrosis may exert cardiovascular pathology, potentially involving chronic inflammation or endothelial dysfunction [31]. Our findings not only reinforce FIB4’s role as a fibrosis biomarker but also position it as a cornerstone tool for cardiovascular risk management in CKD.

Subgroup analyses unveiled heterogeneity in FIB4’s predictive performance for cardiovascular mortality across populations [32]. Enhanced prognostic significance was observed in non-Hispanic Black individuals, sedentary patients, and those aged >60 years, suggesting heightened susceptibility to fibrotic cardiovascular injury in these subgroups. Notably, FIB4 demonstrated stronger predictive capacity in males compared to females, potentially attributable to sex-specific hormonal or metabolic variations. Furthermore, smoking and hypertension status amplified FIB4’s association with cardiovascular mortality, indicating synergistic effects between lifestyle factors, comorbidities, and fibrotic risk [33]. These findings advocate for personalized FIB4 monitoring protocols tailored to patient-specific characteristics. While these subgroup analyses suggest differential susceptibility patterns, the exploratory nature of these findings warrants confirmation in dedicated studies due to potential type I error inflation from multiple testing.

Sensitivity analyses confirmed FIB4’s robustness as an independent predictor of mortality, with persistent significance after multivariable adjustment. Importantly, the attenuation of FIB4’s association with cardiovascular mortality following adjustment for inflammatory markers (e.g., NLR) [34]implies partial mediation through inflammatory pathways. However, the predominance of FIB4’s direct effects supports the hypothesis that hepatic fibrosis directly compromises cardiovascular integrity through pathways like structural remodeling (e.g., arterial stiffening) and metabolic toxicity (e.g., uremic solute accumulation, FGF23 axis dysregulation), notwithstanding the partial mediation indicated by NLR. This attenuated mediation via NLR suggests that systemic inflammation, while contributory, does not fully account for the observed mortality relationships; consequently, anti-inflammatory interventions targeting NLR may modulate but not fundamentally alter FIB4associated death pathways driven primarily by fibrotic organ damage. These mechanistic insights align with the emerging cardiohepatic axis paradigm in CKD progression [35], wherein impaired hepatic function propagates multi-organ crosstalk by disrupting metabolic homeostasis (accelerating vascular calcification, amplifying endothelial dysfunction via toxic metabolite burden) and directly inducing myocardial fibrosis, thereby creating a self-perpetuating cycle of cardio-renal-hepatic deterioration that structurally and metabolically amplifies pre-existing cardiovascular vulnerability beyond inflammatory cascades alone.

Our study possesses several notable strengths. First, the prevalence investigation incorporated 28,231 participants, with the large sample size and high external validity ensuring robust conclusions. We systematically analyzed a valuable cohort of 4,907 CKD patients through extended follow-up (median 84 months), with each participant providing comprehensive clinical profiles and survival data. Second, the data collection framework, meticulously designed and conducted by the NCHS team, demonstrated exceptional reliability after rigorous quality control testing. Third, the application of weighted Cox regression models enhanced the generalizability of our findings. Furthermore, the robustness of these regression models was confirmed through VIF analysis, which verified the absence of significant multicollinearity among the selected covariates, strengthening confidence in the model estimates. Crucially, the deliberate exclusion of participants with baseline viral hepatitis or cirrhosis strengthened internal validity by mitigating confounding from advanced liver disease and isolating FIB4 as an independent systemic risk marker, albeit at the cost of restricting applicability to populations without these conditions. Although the sample size might appear moderate, participants effectively represented diverse sociodemographic strata across the United States from 2005 to 2020, ensuring broad applicability and representativeness of our analytical outcomes. Importantly, we established a specific FIB4 threshold for optimizing cardiovascular and all-cause mortality risk management in CKD patients.

However, several limitations warrant consideration. First, the cross-sectional design inherently lacks longitudinal follow-up data for dynamic assessment. Second, the NCHS survival data were limited to records through 2020, precluding access to more recent follow-up information. Finally, the proportion of cardiovascular mortality events was comparatively low. Additionally, the potential for residual confounding remains. While excluding participants with baseline viral hepatitis or cirrhosis mitigated confounding and isolated FIB4 as an independent systemic risk marker, this approach restricts generalizability to populations without advanced liver disease and may underestimate FIB-4’s true association magnitude with CKD in broader populations. These factors necessitate validation in future large-scale prospective cohort studies (utilizing primary data sources) to strengthen the robustness of the conclusions.

Despite these limitations, our study provides pivotal evidence establishing the Fibrosis-4 index as an independent predictor of all-cause and cardiovascular mortality in CKD patients, offering a low-cost, readily accessible multi-organ risk stratification tool for nephrology practice. Unlike conventional cardiovascular risk models (e.g., Framingham score) with limited applicability in CKD populations, FIB4 quantifies hepatic-renal-cardiovascular crosstalk by integrating age (a shared risk factor for renal/cardiovascular events), platelet count (reflecting inflammatory status), and hepatic transaminases (indicating metabolic/hepatogenic injury). With robust predictive performance (AUC: 0.799–0.801, surpassing most single biomarkers) and clear threshold effects (inflection points: 1.84 for all-cause mortality; 1.74 for CVD mortality), FIB4 effectively supplements existing KDIGO risk stratification systems, particularly valuable in primary care or resource-limited settings. Given the nonlinear risk escalation (all-cause mortality HR = 2.57 when FIB4 > 1.84, CVD mortality HR = 2.85 when FIB4 > 1.74), we propose integrating FIB4 into routine cardiovascular risk management pathways: pending further validation, consideration could be given to intensified FIB4 monitoring (e.g., biannually) in high-risk CKD subgroups—particularly older adults (>60 years), non-Hispanic Black individuals, and those with low physical activity (HR = 1.29–1.57)—alongside the standard annual screening recommended for all CKD patients (defined by eGFR < 60 mL/min/1.73 m² or UACR ≥30 mg/g). FIB4 > 1.35 (Q4) should trigger intensified interventions (e.g., cardio-renal specialty referral, cardiac imaging, and early anti-fibrotic therapies like SGLT2 inhibitors), while patients in the “gray zone” (FIB4 1.74–1.84) warrant closer monitoring (e.g., 6-month reassessment). This approach optimizes resource allocation by prioritizing FIB4 > 1.84 patients for dedicated cardio-renal programs. Importantly, clinical application must account for non-hepatic confounders: ALT/AST elevations may originate from muscle injury, systemic inflammation, or drug toxicity rather than hepatic fibrosis. Although active liver diseases were excluded in this study, such confounders are prevalent in clinical practice. We recommend confirmatory testing with gamma-glutamyl transferase/alkaline phosphatase, creatine kinase (CK), and CRP to differentiate etiologies, with repeat FIB4 measurement after resolving acute events to avoid over-referral. Future studies should validate dynamic FIB4 trajectories across diverse ethnic populations and CKD stages, while developing CKD-specific modified algorithms (e.g., incorporating myoglobin or inflammatory markers) to enhance specificity in non-hepatic conditions. These efforts will ultimately advance precision medicine for high-risk CKD patients.

## Conclusion

Our study demonstrates that elevated FIB4 levels serve as an independent risk factor for both cardiovascular mortality (FIB4 > 1.74) and all-cause mortality (FIB4 > 1.84) in patients with CKD. These findings establish FIB4 as a novel and effective prognostic predictor with broad applicability for risk stratification in CKD patients.

## Supporting information

S1 Table Basic demographic data of CKD and non-CKD subjects. All estimates accounted for complex survey designs. Values are presented as mean ± SD for continuous variables, and P-value was calculated by the weighted linear regression. Values are presented as percent (%) for categorical variables, and P-value was calculated by weighted chi-square test. ALB: albumin; ALT: alanine aminotransferase; AST: aspartate aminotransferase; TG: triglycerides; UA: uric acid; Scr: serum creatinine; FBG: fasting blood glucose; Lym: lymphocyte count; Segne: segmented neutrophils; Plt: platelet count; HDL: high-density lipoprotein; Uscr: urinary creatinine; UACR: urinary albumin-to-creatinine ratio; EGFR: estimated glomerular filtration rate; FIB4: Fibrosis-4 index; NLR: neutrophil-to-lymphocyte ratio; BMI: body mass index; PIR: poverty-income ratio; SD: standard deviation; HR: hazard ratio; CI: confidence interval; OR: odds ratio; t: Student’s t-test; χ²: chi-square test.
(DOCX)

S2 Table Weighted baseline characteristics of all-cause mortality in CKD patients. All estimates accounted for complex survey designs. Values are presented as mean ± SD for continuous variables, and P-value was calculated by the weighted linear regression. Values are presented as percent (%) for categorical variables, and P-value was calculated by weighted chi-square test. ALB: albumin; ALT: alanine aminotransferase; AST: aspartate aminotransferase; TG: triglycerides; UA: uric acid; Scr: serum creatinine; FBG: fasting blood glucose; Lym: lymphocyte count; Segne: segmented neutrophils; Plt: platelet count; HDL: high-density lipoprotein; Uscr: urinary creatinine; UACR: urinary albumin-to-creatinine ratio; EGFR: estimated glomerular filtration rate; FIB4: Fibrosis-4 index; NLR: neutrophil-to-lymphocyte ratio; BMI: body mass index; PIR: poverty-income ratio; SD: standard deviation; HR: hazard ratio; CI: confidence interval; OR: odds ratio; t: Student’s t-test; χ²: chi-square test.
(DOCX)

S3 Table Weighted baseline characteristics of CVD mortality in CKD patients. All estimates accounted for complex survey designs. Values are presented as mean ± SD for continuous variables, and P-value was calculated by the weighted linear regression. Values are presented as percent (%) for categorical variables, and P-value was calculated by weighted chi-square test. ALB: albumin; ALT: alanine aminotransferase; AST: aspartate aminotransferase; TG: triglycerides; UA: uric acid; Scr: serum creatinine; FBG: fasting blood glucose; Lym: lymphocyte count; Segne: segmented neutrophils; Plt: platelet count; HDL: high-density lipoprotein; Uscr: urinary creatinine; UACR: urinary albumin-to-creatinine ratio; EGFR: estimated glomerular filtration rate; FIB4: Fibrosis-4 index; NLR: neutrophil-to-lymphocyte ratio; BMI: body mass index; PIR: poverty-income ratio; SD: standard deviation; HR: hazard ratio; CI: confidence interval; OR: odds ratio; t: Student’s t-test; χ²: chi-square test.
(DOCX)

S4 Table Mediation analysis results of FIB4, NLR and all-cause mortality. CI: Confidence Interval.
(DOCX)

S5 Table Pathway analysis of mediation effects of FIB4, NLR and all-cause mortality. SD: Standard Deviation; CI: Confidence Interval; FIB4: Fibrosis-4 index; NLR: neutrophil-to-lymphocyte ratio.
(DOCX)

S6 Table Mediation effect analysis of FIB4, NLR and CVD mortality. CI: Confidence Interval.
(DOCX)

S7 Table Pathway analysis of mediation effects of FIB4, NLR and CVD mortality. SD: Standard Deviation; CI: Confidence Interval; FIB4: Fibrosis-4 index; NLR: neutrophil-to-lymphocyte ratio; CVD: cardiovascular disease.
(DOCX)

S8 Table VIF detection in logistic regression. GVIF: Generalized Variance Inflation Factor; Df: Degrees of Freedom.
(DOCX)

S9 Table VIF detection in Cox regression. GVIF: Generalized Variance Inflation Factor; Df: Degrees of Freedom.
(DOCX)
