# Supplementary material for: Fibrosis-4 index as a predictor of all-cause and cardiovascular mortality in patients with chronic kidney disease
Source: PLoS One. 2025 Aug 1;20(8):e0329315. doi: 10.1371/journal.pone.0329315 (PMC12316213; doi:10.1371/journal.pone.0329315)
Supplement: S1 Table — All estimates accounted for complex survey designs. Values are presented as mean ± SD for continuous variables, and P-value was calculated by the weighted linear regression. Values are presented as percent (%) for categorical variables, and P-value was calculated by weighted chi-square test. ALB: albumin; ALT: alanine aminotransferase; AST: aspartate aminotransferase; TG: triglycerides; UA: uric acid; Scr: serum creatinine; FBG: fasting blood glucose; Lym: lymphocyte count; Segne: segmented neutrophils; Plt: platelet count; HDL: high-density lipoprotein; Uscr: urinary creatinine; UACR: urinary albumin-to-creatinine ratio; EGFR: estimated glomerular filtration rate; FIB4: Fibrosis-4 index; NLR: neutrophil-to-lymphocyte ratio; BMI: body mass index; PIR: poverty-income ratio; SD: standard deviation; HR: hazard ratio; CI: confidence interval; OR: odds ratio; t: Student’s t-test; χ²: chi-square test. (DOCX) [file pone.0329315.s001.docx]

| Variable | Total  (n = 28231) | non-CKD  (n=23324) | CKD  (n=4907) | Statistic | *P* |
| --- | --- | --- | --- | --- | --- |
|  |  |  |  |  |  |
| ALB, Mean (SD) | 4.29 (0.00) | 4.31 (0.00) | 4.19 (0.01) | t=-15.32 | <.001 |
| ALT, Mean (SD) | 25.28 (0.14) | 25.50 (0.15) | 23.90 (0.40) | t=-3.82 | <.001 |
| AST, Mean (SD) | 25.42 (0.11) | 25.30 (0.11) | 26.17 (0.31) | t=2.70 | 0.008 |
| TG, Mean (SD) | 152.91 (1.31) | 149.91 (1.41) | 171.53 (2.68) | t=7.41 | <.001 |
| UA, Mean (SD) | 5.40 (0.01) | 5.31 (0.01) | 5.96 (0.03) | t=20.35 | <.001 |
| Scr, Mean (SD) | 78.24 (0.23) | 74.99 (0.19) | 98.41 (1.06) | t=22.04 | <.001 |
| FBG, Mean (SD) | 5.59 (0.01) | 5.50 (0.01) | 6.12 (0.03) | t=23.72 | <.001 |
| Lym, Mean (SD) | 2.13 (0.01) | 2.14 (0.01) | 2.07 (0.02) | t=-3.51 | <.001 |
| Segne, Mean (SD) | 4.32 (0.02) | 4.28 (0.02) | 4.60 (0.04) | t=9.65 | <.001 |
| Plt, Mean (SD) | 249.39 (0.79) | 250.29 (0.83) | 243.76 (1.39) | t=-4.58 | <.001 |
| HDL, Mean (SD) | 1.39 (0.01) | 1.39 (0.01) | 1.38 (0.01) | t=-0.66 | 0.513 |
| Uscr, Mean (SD) | 120.55 (0.89) | 121.79 (0.97) | 112.85 (1.31) | t=-5.98 | <.001 |
| BMI, Mean (SD) | 28.78 (0.08) | 28.61 (0.09) | 29.88 (0.15) | t=8.94 | <.001 |
| FIB4, Mean (SD) | 1.08 (0.01) | 1.00 (0.01) | 1.56 (0.02) | t=30.00 | <.001 |
| UACR, Mean (SD) | 31.30 (1.44) | 7.71 (0.06) | 177.75 (10.28) | t=16.54 | <.001 |
| EGFR, Mean (SD) | 99.86 (0.30) | 103.75 (0.25) | 75.70 (0.61) | t=-52.95 | <.001 |
| NLR, Mean (SD) | 2.20 (0.01) | 2.15 (0.01) | 2.53 (0.03) | t=15.20 | <.001 |
| Sex, n(%) |  |  |  | χ²=70.53 | <.001 |
| Male | 13661 (48.12) | 11362 (49.12) | 2299 (41.90) |  |  |
| Female | 14570 (51.88) | 11962 (50.88) | 2608 (58.10) |  |  |
| Ethnicity, n(%) |  |  |  | χ²=36.40 | <.001 |
| Mexican American | 4617 (8.56) | 3936 (8.77) | 681 (7.25) |  |  |
| Other Hispanic | 2695 (5.29) | 2304 (5.43) | 391 (4.44) |  |  |
| Non-Hispanic White | 12271 (68.17) | 9906 (67.89) | 2365 (69.96) |  |  |
| Non-Hispanic Black | 5846 (10.87) | 4732 (10.62) | 1114 (12.42) |  |  |
| Other Race | 2802 (7.10) | 2446 (7.29) | 356 (5.93) |  |  |
| Marital status, n(%) |  |  |  | χ²=64.77 | <.001 |
| Married | 17103 (64.38) | 14420 (65.30) | 2683 (58.67) |  |  |
| Other (widowed, divorced, Separated, never married, living with a partner) | 11128 (35.62) | 8904 (34.70) | 2224 (41.33) |  |  |
| PIR, n(%) |  |  |  | χ²=9.00 | 0.008 |
| Poor | 5513 (13.24) | 4518 (13.00) | 995 (14.75) |  |  |
| Not Poor | 22718 (86.76) | 18806 (87.00) | 3912 (85.25) |  |  |
| Smoking, n(%) |  |  |  | χ²=29.77 | <.001 |
| No | 15767 (55.32) | 13270 (55.97) | 2497 (51.30) |  |  |
| Yes | 12464 (44.68) | 10054 (44.03) | 2410 (48.70) |  |  |
| Education level, n(%) |  |  |  | χ²=202.39 | <.001 |
| Less than high school | 7192 (16.70) | 5589 (15.65) | 1603 (23.26) |  |  |
| high school or equivalent | 6448 (22.48) | 5250 (22.01) | 1198 (25.44) |  |  |
| college or above | 14591 (60.81) | 12485 (62.35) | 2106 (51.30) |  |  |
| Drinking, n(%) |  |  |  | χ²=115.15 | <.001 |
| No | 9738 (28.65) | 7768 (27.49) | 1970 (35.84) |  |  |
| Yes | 18493 (71.35) | 15556 (72.51) | 2937 (64.16) |  |  |
| Physical activity, n(%) |  |  |  | χ²=457.57 | <.001 |
| Low physical activity | 11849 (37.53) | 9054 (35.06) | 2795 (52.89) |  |  |
| High physical activity | 16382 (62.47) | 14270 (64.94) | 2112 (47.11) |  |  |
| Hypertension, n(%) |  |  |  | χ²=1777.10 | <.001 |
| No | 16866 (64.11) | 15428 (68.94) | 1438 (34.13) |  |  |
| Yes | 11365 (35.89) | 7896 (31.06) | 3469 (65.87) |  |  |
| **Diabetes mellitus**, n(%) |  |  |  | χ²=1669.51 | <.001 |
| No | 23261 (86.84) | 20307 (90.14) | 2954 (66.36) |  |  |
| Yes | 4970 (13.16) | 3017 (9.86) | 1953 (33.64) |  |  |
| Age, n(%) |  |  |  | χ²=2898.50 | <.001 |
| <=60 | 19306 (75.39) | 17633 (80.93) | 1673 (41.00) |  |  |
| >60 | 8925 (24.61) | 5691 (19.07) | 3234 (59.00) |  |  |
