# Supplementary material for: Fibrosis-4 index as a predictor of all-cause and cardiovascular mortality in patients with chronic kidney disease
Source: PLoS One. 2025 Aug 1;20(8):e0329315. doi: 10.1371/journal.pone.0329315 (PMC12316213; doi:10.1371/journal.pone.0329315)
Supplement: S2 Table — All estimates accounted for complex survey designs. Values are presented as mean ± SD for continuous variables, and P-value was calculated by the weighted linear regression. Values are presented as percent (%) for categorical variables, and P-value was calculated by weighted chi-square test. ALB: albumin; ALT: alanine aminotransferase; AST: aspartate aminotransferase; TG: triglycerides; UA: uric acid; Scr: serum creatinine; FBG: fasting blood glucose; Lym: lymphocyte count; Segne: segmented neutrophils; Plt: platelet count; HDL: high-density lipoprotein; Uscr: urinary creatinine; UACR: urinary albumin-to-creatinine ratio; EGFR: estimated glomerular filtration rate; FIB4: Fibrosis-4 index; NLR: neutrophil-to-lymphocyte ratio; BMI: body mass index; PIR: poverty-income ratio; SD: standard deviation; HR: hazard ratio; CI: confidence interval; OR: odds ratio; t: Student’s t-test; χ²: chi-square test. (DOCX) [file pone.0329315.s002.docx]

| Variable | Total (n = 4907) | CKD patients alive  (n=3345) | CKD patients dead  (n=1562) | Statistic | *P* |
| --- | --- | --- | --- | --- | --- |
|  |  |  |  |  |  |
| ALB, Mean (SD) | 4.19 (0.01) | 4.23 (0.01) | 4.09 (0.01) | t=-9.51 | **<.001** |
| ALT, Mean (SD) | 23.90 (0.40) | 24.35 (0.38) | 22.68 (1.04) | t=-1.52 | 0.131 |
| AST, Mean (SD) | 26.17 (0.31) | 25.76 (0.27) | 27.28 (0.84) | t=1.75 | 0.084 |
| TG, Mean (SD) | 171.53 (2.68) | 172.83 (3.13) | 167.98 (4.26) | t=-0.98 | 0.329 |
| UA, Mean (SD) | 5.96 (0.03) | 5.81 (0.04) | 6.36 (0.05) | t=9.22 | **<.001** |
| Scr, Mean (SD) | 98.41 (1.06) | 91.90 (0.94) | 116.12 (2.41) | t=10.03 | **<.001** |
| FBG, Mean (SD) | 6.12 (0.03) | 6.08 (0.03) | 6.21 (0.04) | t=2.87 | **0.005** |
| Lym, Mean (SD) | 2.07 (0.02) | 2.09 (0.02) | 1.99 (0.06) | t=-1.44 | 0.154 |
| Segne, Mean (SD) | 4.60 (0.04) | 4.50 (0.04) | 4.84 (0.07) | t=4.55 | **<.001** |
| Plt, Mean (SD) | 243.76 (1.39) | 246.77 (1.60) | 235.55 (2.76) | t=-3.60 | **<.001** |
| HDL, Mean (SD) | 1.38 (0.01) | 1.38 (0.01) | 1.38 (0.02) | t=-0.01 | 0.989 |
| Uscr, Mean (SD) | 112.85 (1.31) | 116.46 (1.73) | 103.04 (2.06) | t=-4.73 | **<.001** |
| BMI, Mean (SD) | 29.88 (0.15) | 30.05 (0.19) | 29.42 (0.23) | t=-2.12 | **0.037** |
| FIB4, Mean (SD) | 1.56 (0.02) | 1.36 (0.02) | 2.08 (0.05) | t=12.51 | **<.001** |
| UACR, Mean (SD) | 177.75 (10.28) | 149.44 (10.53) | 254.75 (25.84) | t=3.73 | **<.001** |
| EGFR, Mean (SD) | 75.70 (0.61) | 81.41 (0.74) | 60.16 (0.79) | t=-19.80 | **<.001** |
| NLR, Mean (SD) | 2.53 (0.03) | 2.37 (0.02) | 3.00 (0.06) | t=9.45 | **<.001** |
| Sex, n(%) |  |  |  | χ²=15.14 | **0.002** |
| Male | 2299 (41.90) | 1449 (40.23) | 850 (46.41) |  |  |
| Female | 2608 (58.10) | 1896 (59.77) | 712 (53.59) |  |  |
| Ethnicity, n(%) |  |  |  | χ²=86.73 | **<.001** |
| Mexican American | 681 (7.25) | 550 (8.53) | 131 (3.77) |  |  |
| Other Hispanic | 391 (4.44) | 320 (5.32) | 71 (2.04) |  |  |
| Non-Hispanic White | 2365 (69.96) | 1388 (66.66) | 977 (78.95) |  |  |
| Non-Hispanic Black | 1114 (12.42) | 794 (12.90) | 320 (11.11) |  |  |
| Other Race | 356 (5.93) | 293 (6.60) | 63 (4.14) |  |  |
| Marital status, n(%) |  |  |  | χ²=64.20 | **<.001** |
| Married | 2683 (58.67) | 1932 (62.08) | 751 (49.38) |  |  |
| Other (widowed, divorced, separated, never married, living with a partner) | 2224 (41.33) | 1413 (37.92) | 811 (50.62) |  |  |
| PIR, n(%) |  |  |  | χ²=1.47 | 0.316 |
| Poor | 995 (14.75) | 711 (15.13) | 284 (13.74) |  |  |
| Not Poor | 3912 (85.25) | 2634 (84.87) | 1278 (86.26) |  |  |
| Smoking, n(%) |  |  |  | χ²=43.11 | **<.001** |
| No | 2497 (51.30) | 1829 (54.14) | 668 (43.57) |  |  |
| Yes | 2410 (48.70) | 1516 (45.86) | 894 (56.43) |  |  |
| Education level, n(%) |  |  |  | χ²=79.64 | **<.001** |
| Less than high school | 1603 (23.26) | 1006 (20.41) | 597 (31.01) |  |  |
| high school or equivalent | 1198 (25.44) | 803 (24.84) | 395 (27.09) |  |  |
| college or above | 2106 (51.30) | 1536 (54.76) | 570 (41.90) |  |  |
| Drinking, n(%) |  |  |  | χ²=25.86 | **<.001** |
| No | 1970 (35.84) | 1309 (33.73) | 661 (41.58) |  |  |
| Yes | 2937 (64.16) | 2036 (66.27) | 901 (58.42) |  |  |
| Physical activity, n(%) |  |  |  | χ²=220.25 | **<.001** |
| Low physical activity | 2795 (52.89) | 1681 (46.48) | 1114 (70.33) |  |  |
| High physical activity | 2112 (47.11) | 1664 (53.52) | 448 (29.67) |  |  |
| Hypertension, n(%) |  |  |  | χ²=231.28 | **<.001** |
| No | 1438 (34.13) | 1159 (40.37) | 279 (17.16) |  |  |
| Yes | 3469 (65.87) | 2186 (59.63) | 1283 (82.84) |  |  |
| **Diabetes mellitus**, n(%) |  |  |  | χ²=82.98 | **<.001** |
| No | 2954 (66.36) | 2127 (70.09) | 827 (56.23) |  |  |
| Yes | 1953 (33.64) | 1218 (29.91) | 735 (43.77) |  |  |
| Age, n(%) |  |  |  | χ²=612.93 | **<.001** |
| <=60 | 1673 (41.00) | 1527 (51.54) | 146 (12.33) |  |  |
| >60 | 3234 (59.00) | 1818 (48.46) | 1416 (87.67) |  |  |
