# Supplementary material for: Fibrosis-4 index as a predictor of all-cause and cardiovascular mortality in patients with chronic kidney disease
Source: PLoS One. 2025 Aug 1;20(8):e0329315. doi: 10.1371/journal.pone.0329315 (PMC12316213; doi:10.1371/journal.pone.0329315)
Supplement: S3 Table — All estimates accounted for complex survey designs. Values are presented as mean ± SD for continuous variables, and P-value was calculated by the weighted linear regression. Values are presented as percent (%) for categorical variables, and P-value was calculated by weighted chi-square test. ALB: albumin; ALT: alanine aminotransferase; AST: aspartate aminotransferase; TG: triglycerides; UA: uric acid; Scr: serum creatinine; FBG: fasting blood glucose; Lym: lymphocyte count; Segne: segmented neutrophils; Plt: platelet count; HDL: high-density lipoprotein; Uscr: urinary creatinine; UACR: urinary albumin-to-creatinine ratio; EGFR: estimated glomerular filtration rate; FIB4: Fibrosis-4 index; NLR: neutrophil-to-lymphocyte ratio; BMI: body mass index; PIR: poverty-income ratio; SD: standard deviation; HR: hazard ratio; CI: confidence interval; OR: odds ratio; t: Student’s t-test; χ²: chi-square test. (DOCX) [file pone.0329315.s003.docx]

| Variable | Total (n = 4907) | 0 (n=4340) | 1 (n=567) | Statistic | *P* |
| --- | --- | --- | --- | --- | --- |
|  |  |  |  |  |  |
| ALB, Mean (SD) | 4.19 (0.01) | 4.20 (0.01) | 4.08 (0.02) | t=-5.59 | <.001 |
| ALT, Mean (SD) | 23.90 (0.40) | 24.17 (0.43) | 21.37 (0.62) | t=-3.73 | <.001 |
| AST, Mean (SD) | 26.17 (0.31) | 26.15 (0.34) | 26.35 (0.48) | t=0.35 | 0.728 |
| TG, Mean (SD) | 171.53 (2.68) | 172.19 (2.91) | 165.15 (4.85) | t=-1.25 | 0.215 |
| UA, Mean (SD) | 5.96 (0.03) | 5.90 (0.03) | 6.47 (0.09) | t=6.04 | <.001 |
| Scr, Mean (SD) | 98.41 (1.06) | 96.55 (1.12) | 116.22 (2.99) | t=6.02 | <.001 |
| FBG, Mean (SD) | 6.12 (0.03) | 6.11 (0.03) | 6.20 (0.06) | t=1.45 | 0.151 |
| Lym, Mean (SD) | 2.07 (0.02) | 2.09 (0.02) | 1.86 (0.04) | t=-4.97 | <.001 |
| Segne, Mean (SD) | 4.60 (0.04) | 4.58 (0.04) | 4.79 (0.09) | t=2.19 | 0.031 |
| Plt, Mean (SD) | 243.76 (1.39) | 244.37 (1.42) | 237.88 (5.27) | t=-1.20 | 0.232 |
| HDL, Mean (SD) | 1.38 (0.01) | 1.39 (0.01) | 1.34 (0.02) | t=-2.15 | 0.034 |
| Uscr, Mean (SD) | 112.85 (1.31) | 113.66 (1.48) | 105.04 (3.01) | t=-2.41 | 0.018 |
| BMI, Mean (SD) | 29.88 (0.15) | 29.89 (0.16) | 29.84 (0.41) | t=-0.09 | 0.928 |
| FIB4, Mean (SD) | 1.56 (0.02) | 1.50 (0.02) | 2.08 (0.05) | t=9.47 | <.001 |
| UACR, Mean (SD) | 177.75 (10.28) | 170.00 (10.07) | 252.11 (47.90) | t=1.68 | 0.095 |
| EGFR, Mean (SD) | 75.70 (0.61) | 77.47 (0.65) | 58.72 (1.22) | t=-12.93 | <.001 |
| NLR, Mean (SD) | 2.53 (0.03) | 2.49 (0.03) | 3.00 (0.09) | t=4.97 | <.001 |
| Sex, n(%) |  |  |  | χ²=5.74 | 0.021 |
| Male | 2299 (41.90) | 1992 (41.35) | 307 (47.12) |  |  |
| Female | 2608 (58.10) | 2348 (58.65) | 260 (52.88) |  |  |
| Ethnicity, n(%) |  |  |  | χ²=24.28 | <.001 |
| Mexican American | 681 (7.25) | 642 (7.62) | 39 (3.64) |  |  |
| Other Hispanic | 391 (4.44) | 363 (4.65) | 28 (2.38) |  |  |
| Non-Hispanic White | 2365 (69.96) | 2016 (69.14) | 349 (77.87) |  |  |
| Non-Hispanic Black | 1114 (12.42) | 980 (12.39) | 134 (12.73) |  |  |
| Other Race | 356 (5.93) | 339 (6.20) | 17 (3.38) |  |  |
| Marital status, n(%) |  |  |  | χ²=21.24 | <.001 |
| Married | 2683 (58.67) | 2423 (59.71) | 260 (48.63) |  |  |
| Other (widowed, divorced, separated, never married, living with a partner) | 2224 (41.33) | 1917 (40.29) | 307 (51.37) |  |  |
| PIR, n(%) |  |  |  | χ²=0.49 | 0.560 |
| Poor | 995 (14.75) | 898 (14.87) | 97 (13.66) |  |  |
| Not Poor | 3912 (85.25) | 3442 (85.13) | 470 (86.34) |  |  |
| Smoking, n(%) |  |  |  | χ²=2.31 | 0.208 |
| No | 2497 (51.30) | 2220 (51.65) | 277 (47.94) |  |  |
| Yes | 2410 (48.70) | 2120 (48.35) | 290 (52.06) |  |  |
| Education level, n(%) |  |  |  | χ²=18.89 | <.001 |
| Less than high school | 1603 (23.26) | 1398 (22.63) | 205 (29.31) |  |  |
| high school or equivalent | 1198 (25.44) | 1054 (25.10) | 144 (28.72) |  |  |
| college or above | 2106 (51.30) | 1888 (52.27) | 218 (41.97) |  |  |
| Drinking, n(%) |  |  |  | χ²=12.28 | <.001 |
| No | 1970 (35.84) | 1725 (35.06) | 245 (43.27) |  |  |
| Yes | 2937 (64.16) | 2615 (64.94) | 322 (56.73) |  |  |
| Physical activity, n(%) |  |  |  | χ²=63.40 | <.001 |
| Low physical activity | 2795 (52.89) | 2398 (51.06) | 397 (70.46) |  |  |
| High physical activity | 2112 (47.11) | 1942 (48.94) | 170 (29.54) |  |  |
| Hypertension, n(%) |  |  |  | χ²=78.10 | <.001 |
| No | 1438 (34.13) | 1342 (36.06) | 96 (15.61) |  |  |
| Yes | 3469 (65.87) | 2998 (63.94) | 471 (84.39) |  |  |
| **Diabetes mellitus**, n(%) |  |  |  | χ²=25.32 | <.001 |
| No | 2954 (66.36) | 2649 (67.46) | 305 (55.85) |  |  |
| Yes | 1953 (33.64) | 1691 (32.54) | 262 (44.15) |  |  |
| Age, n(%) |  |  |  | χ²=211.09 | <.001 |
| <=60 | 1673 (41.00) | 1626 (44.29) | 47 (9.41) |  |  |
| >60 | 3234 (59.00) | 2714 (55.71) | 520 (90.59) |  |  |
