# Supplementary material for: Fibrosis-4 index as a predictor of all-cause and cardiovascular mortality in patients with chronic kidney disease
Source: PLoS One. 2025 Aug 1;20(8):e0329315. doi: 10.1371/journal.pone.0329315 (PMC12316213; doi:10.1371/journal.pone.0329315)
Supplement: S4 Table — CI: Confidence Interval. (DOCX) [file pone.0329315.s004.docx]

| Effect | Estimate | Lower | Upper | β (95% CI) | P-value | Proportion |
| --- | --- | --- | --- | --- | --- | --- |
| Indirect | -1.48 | -2.12 | -0.89 | -1.48 (-2.12 to -0.89) | <0.001 | 5.48 |
| Direct | -25.99 | -33.61 | -18.69 | -25.99 (-33.61 to -18.69) | <0.001 | 94.52 |
| Total | -27.48 | -34.86 | -19.97 | -27.48 (-34.86 to -19.97) | <0.001 | 100.00 |
