# Supplementary material for: Fibrosis-4 index as a predictor of all-cause and cardiovascular mortality in patients with chronic kidney disease
Source: PLoS One. 2025 Aug 1;20(8):e0329315. doi: 10.1371/journal.pone.0329315 (PMC12316213; doi:10.1371/journal.pone.0329315)
Supplement: S5 Table — SD: Standard Deviation; CI: Confidence Interval; FIB4: Fibrosis-4 index; NLR: neutrophil-to-lymphocyte ratio. (DOCX) [file pone.0329315.s005.docx]

| Pathway | β | SD | Lower | Upper | P-value | β (95% CI) |
| --- | --- | --- | --- | --- | --- | --- |
| FIB4 → NLR | 0.12 | 0.03 | 0.06 | 0.18 | <0.001 | 0.12 (0.06-0.18) |
| FIB4 →all-cause mortality | 0.04 | 0.04 | -0.18 | 0.34 | 0.018 | 0.04 (-0.18-0.34) |
| NLR →all-cause mortality | 0.22 | 0.02 | 0.18 | 0.26 | <0.001 | 0.22 (0.18-0.26) |
