# Supplementary material for: Fibrosis-4 index as a predictor of all-cause and cardiovascular mortality in patients with chronic kidney disease
Source: PLoS One. 2025 Aug 1;20(8):e0329315. doi: 10.1371/journal.pone.0329315 (PMC12316213; doi:10.1371/journal.pone.0329315)
Supplement: S6 Table — CI: Confidence Interval. (DOCX) [file pone.0329315.s006.docx]

| Effect | Estimate | Lower | Upper | β (95% CI) | P-value | Proportion |
| --- | --- | --- | --- | --- | --- | --- |
| Indirect | -1.54 | -2.23 | -0.91 | -1.54 (-2.23 to -0.91) | <0.001 | 5.83 |
| Direct | -24.89 | -33.11 | -16.01 | -24.89 (-33.11 to -16.01) | <0.001 | 94.17 |
| Total | -26.43 | -34.42 | -17.34 | -26.43 (-34.42 to -17.34) | <0.001 | 100.00 |
