# Supplementary material for: Fibrosis-4 index as a predictor of all-cause and cardiovascular mortality in patients with chronic kidney disease
Source: PLoS One. 2025 Aug 1;20(8):e0329315. doi: 10.1371/journal.pone.0329315 (PMC12316213; doi:10.1371/journal.pone.0329315)
Supplement: S7 Table — SD: Standard Deviation; CI: Confidence Interval; FIB4: Fibrosis-4 index; NLR: neutrophil-to-lymphocyte ratio; CVD: cardiovascular disease. (DOCX) [file pone.0329315.s007.docx]

| Pathway | β | SD | Lower | Upper | P-value | β (95% CI) |
| --- | --- | --- | --- | --- | --- | --- |
| FIB4 → NLR | 0.12 | 0.03 | 0.06 | 0.18 | <0.001 | 0.12 (0.06-0.18) |
| FIB4 →CVD mortality | 0.26 | 0.05 | 0.16 | 0.36 | <0.001 | 0.26 (0.16-0.36) |
| NLR →CVD mortality | 0.22 | 0.03 | 0.17 | 0.27 | <0.001 | 0.22 (0.17-0.27) |
