# Supplementary material for: Fibrosis-4 index as a predictor of all-cause and cardiovascular mortality in patients with chronic kidney disease
Source: PLoS One. 2025 Aug 1;20(8):e0329315. doi: 10.1371/journal.pone.0329315 (PMC12316213; doi:10.1371/journal.pone.0329315)
Supplement: S8 Table — GVIF: Generalized Variance Inflation Factor; Df: Degrees of Freedom. (DOCX) [file pone.0329315.s008.docx]

| Variable | GVIF | Df | GVIF^(1/(2*Df)) |
| --- | --- | --- | --- |
| FIB4 | 1.755 | 1 | 1.325 |
| Sex | 1.135 | 1 | 1.065 |
| Ethnicity | 1.306 | 4 | 1.034 |
| Marital status | 1.062 | 1 | 1.031 |
| PIR | 1.122 | 1 | 1.059 |
| Smoking | 1.151 | 1 | 1.073 |
| Education level | 1.273 | 2 | 1.062 |
| Drinking | 1.18 | 1 | 1.086 |
| Physical activity | 1.094 | 1 | 1.046 |
| Hypertension | 1.376 | 1 | 1.173 |
| Diabetes mellitus | 1.166 | 1 | 1.08 |
