# Supplementary material for: Fibrosis-4 index as a predictor of all-cause and cardiovascular mortality in patients with chronic kidney disease
Source: PLoS One. 2025 Aug 1;20(8):e0329315. doi: 10.1371/journal.pone.0329315 (PMC12316213; doi:10.1371/journal.pone.0329315)
Supplement: S9 Table — GVIF: Generalized Variance Inflation Factor; Df: Degrees of Freedom. (DOCX) [file pone.0329315.s009.docx]

| Variable | GVIF | Df | GVIF^(1/(2*Df)) |
| --- | --- | --- | --- |
| FIB4 | 1.459 | 1 | 1.208 |
| Sex | 1.217 | 1 | 1.103 |
| Ethnicity | 1.36 | 4 | 1.039 |
| Marital status | 1.108 | 1 | 1.053 |
| PIR | 1.129 | 1 | 1.063 |
| Smoking | 1.158 | 1 | 1.076 |
| Education level | 1.209 | 2 | 1.049 |
| Drinking | 1.208 | 1 | 1.099 |
| Physical activity | 1.1 | 1 | 1.049 |
| Hypertension | 1.239 | 1 | 1.113 |
| Diabetes mellitus | 1.1 | 1 | 1.049 |
